# Evaluating a Modified American Society of Anesthesiologists Physical Status‐Based Sedation Management System for Pancreatobiliary Endoscopy: A Prospective Feasibility Study

**DOI:** 10.1002/deo2.70445

**Published:** 2026-10-12

**Authors:** Taro Hanaoka, Kosuke Okuwaki, Masafumi Watanabe, Hiromi Matsuda, Kai Adachi, Akihiro Tamaki, Daisuke Ogawa, Mayuko Sakai, Chika Kusano

**Affiliations:** ^1^ Department of Gastroenterology Kitasato University School of Medicine Kanagawa Japan; ^2^ Department of Anesthesiology Kitasato University School of Medicine Kanagawa Japan

**Keywords:** anesthesiologists, endoscopy, pancreaticobiliary system, procedural sedation, risk assessment

## Abstract

**Objectives:**

To prospectively evaluate the feasibility of implementing a modified American Society of Anesthesiologists Physical Status (ASA‐PS)‐based sedation management system for endoscopist‐managed sedation in pancreatobiliary endoscopy.

**Methods:**

This single‐center, single‐arm prospective feasibility study involved 2000 patients (2024–2025). Sedation risk was stratified using a modified ASA‐PS classification system combined with predefined high‐risk clinical factors (severe obesity, obstructive sleep apnea requiring continuous positive airway pressure, chronic obstructive pulmonary disease requiring home oxygen therapy, reduced functional capacity, and difficult sedation history). Patients meeting high‐risk criteria underwent pre‐procedural anesthesiologist assessment to determine sedation providers. The primary outcome was sedation‐related adverse event (SRAE) incidence during endoscopist‐managed sedation. The secondary outcomes were the sedation success rate and SRAE‐associated risk factors.

**Results:**

Among 2000 patients, 231 (11.6%) were classified as having high risk for sedation. Post‐assessment, anesthesiologists and endoscopists managed sedation for 86 (4.3%) and 1914 (95.7%) patients, respectively. In the endoscopist‐managed group, SRAEs occurred in 63 patients (3.3%), corresponding to a success rate of 96.7%. No patient required tracheal intubation or conversion to general anesthesia. In multivariable analysis, endoscopic ultrasonography‐guided sampling was independently associated with reduced SRAEs (odds ratio 0.407, 95% confidence interval 0.184–0.899; *p* = 0.026); no independent predictors of increased SRAEs were identified.

**Conclusions:**

The modified ASA‐PS‐based sedation management system may be a feasible approach in settings with limited anesthesiology resources, warranting further validation.

**Trial Registration:**

UMIN000053211

## Graphical abstract

A modified ASA‐PS‐based sedation management system is evaluated in a prospective feasibility study of 2,000 patients undergoing pancreatobiliary endoscopy. Patients are stratified by modified ASA‐PS classification and predefined high‐risk criteria, with anesthesiologist assessment determining sedation providers. Endoscopist‐managed sedation is associated with a low incidence of sedation‐related adverse events (3.3%), supporting the feasibility of this triage approach in settings with limited anesthesiology resources.

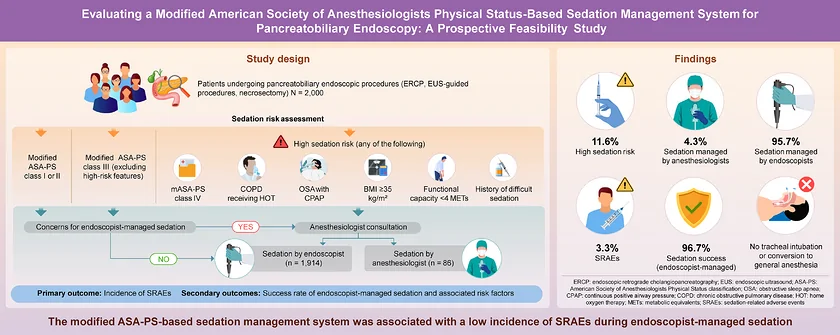

## 1 Introduction

Sedation is a fundamental component of gastrointestinal endoscopy because it alleviates patient discomfort and anxiety while facilitating improved procedural completion and overall quality of care [1, 2]. Advanced endoscopic procedures—endoscopic retrograde cholangiopancreatography (ERCP) and interventional endoscopic ultrasonography (EUS)—are more invasive and technically complex than routine screening endoscopy, requiring larger‐caliber endoscopes and prolonged interventions [3]. Consequently, safe and appropriate sedation management is crucial in these settings. These procedures are frequently performed in older patients and those with substantial comorbidities, in whom sedation‐related risks of respiratory and cardiovascular adverse events are high [3].

The American Society of Anesthesiologists Physical Status (ASA‐PS) classification is a common practical tool for assessing the overall physical status for safe sedation [3, 4]. According to the American Society for Gastrointestinal Endoscopy (ASGE) and ASA guidelines, ASA‐PS Class ≥ IV patients are considered to be at high risk for sedation‐related adverse events (SRAEs), necessitating anesthesiologist‐managed sedation [5, 6]. Additionally, the European Society of Anesthesiology guidelines indicate ASA‐PS Class ≥ III patients as having a high risk for sedation‐related complications [7].

In Western countries, sedation for highly invasive endoscopic procedures is led by anesthesiologists. In contrast, Japan has limited anesthesiology resources, prompting sedation administration and monitoring by endoscopists [3]; therefore, distinguishing patients who can be safely managed by endoscopists from those who require anesthesiologist‐led sedation is essential. In collaboration with anesthesiologists, we developed a modified ASA‐PS‐based sedation management system for patients undergoing pancreatobiliary endoscopic procedures. This system integrates ASA‐PS‐based risk stratification with predefined clinical factors (obesity, respiratory comorbidities, reduced functional capacity, and difficult sedation history) to guide pre‐procedural consultation and sedation provider assignment. This comprises two sequential components—an objective risk‐stratification step based on the modified ASA‐PS classification and predefined high‐risk criteria, and a subsequent anesthesiologist assessment in which the final sedation‐provider decision is made at the anesthesiologist's clinical discretion rather than according to fixed numerical criteria. Here, we aimed to prospectively evaluate the feasibility of implementing the sedation management system in patients undergoing pancreatobiliary endoscopy.

## 2 Methods

### 2.1 Study Design and Outcomes

This was a single‐center, single‐arm prospective feasibility study. The primary outcome was the incidence of SRAEs during endoscopist‐managed sedation. The secondary outcomes were the success rate of endoscopist‐managed sedation; the identification of risk factors for SRAEs; and additional safety‐ and recovery‐related measures, including the requirement for Rapid Response Team activation, procedure completion rate (the complement of the rate of interruption or premature termination of sedation), and modified Aldrete recovery score.

### 2.2 Patient Eligibility

Between January 2024 and October 2025, patients who underwent pancreatobiliary endoscopy at Kitasato University Hospital and met the study eligibility criteria were prospectively enrolled, yielding 2000 eligible cases. The sample size was based on the number of cases expected during the study period rather than a priori power calculation. Eligible procedures included ERCP, balloon enteroscopy‐assisted ERCP, interventional EUS, and endoscopic necrosectomy.

Patients undergoing prospective sedation‐risk assessment using the modified ASA‐PS classification system before the procedure were included; the sole exclusion criterion was the absence of this assessment. Risk assessment was performed at scheduling and reassessed if the patient's condition worsened, with the sedation strategy adjusted accordingly, including anesthesiology consultation when indicated.

### 2.3 Modified ASA‐PS Classification

The modified ASA‐PS was based on the 2000 revised ASA‐PS classification and tailored for practical implementation within the sedation management system for pancreatobiliary endoscopy (Table 1), with key modifications including disease entities directly relevant to endoscopic sedation risk (e.g., obstructive jaundice and severe cholangitis) and redefined morbid obesity. These modifications and the high‐risk criteria were not derived from published evidence but established through institutional experience and consensus among endoscopists and anesthesiologists. They were approved by the institutional risk management committee (which includes both specialties), aiming to operationalize ASA‐PS for sedation‐provider decision‐making rather than redefine perioperative risk. Sedation‐specific high‐risk features (functional capacity <4 metabolic equivalents (METs) and difficult sedation history) were also predefined as independent triage criteria.

**TABLE 1:** Modified American Society of Anesthesiologists Physical Status (ASA‐PS) classification used for sedation risk assessment.

Class	Definition	Examples
I	A normal healthy patient	Healthy, non‐smoking, no or minimal alcohol use
II	A patient with mild systemic disease	Current smoker, social alcohol drinker, pregnancy, obesity (BMI <35), mild lung disease, obstructive jaundice, OSA without CPAP
III	A patient with severe systemic disease	COPD receiving HOT, OSA with CPAP, morbid obesity (BMI ≥35), active hepatitis, alcohol dependence or abuse, implanted pacemaker, ESRD undergoing regularly scheduled dialysis, history (>3 months) of MI, Others
IV	A patient with severe systemic disease that is a constant threat to life	Recent (<3 months) MI, CVA, TIA or CAD/stents, ongoing cardiac ischemia or severe valve dysfunction, shock, sepsis, DIC, severe cholangitis, ARD or ESRD not undergoing regularly scheduled dialysis
Sedation‐specific high‐risk features		Functional capacity <4 METs, History of difficult sedation

Abbreviations: BMI, body mass index; OSA, obstructive sleep apnea; CPAP, continuous positive airway pressure; COPD, chronic obstructive pulmonary disease; HOT, home oxygen therapy; ESRD, end‐stage renal disease; MI, myocardial infarction; CVA, cerebrovascular accident; TIA, transient ischemic attack; CAD, coronary artery disease; ARD, acute renal dysfunction; METs, metabolic equivalents.

### 2.4 Sedation Risk Assessment

Based on the modified ASA‐PS classification system combined with predefined sedation‐specific high‐risk features, patients were classified as having high risk for sedation if they met any of the following criteria: modified ASA‐PS Class IV; modified ASA‐PS Class III with morbid obesity (body mass index ≥35 kg/m^2^), obstructive sleep apnea requiring continuous positive airway pressure, or chronic obstructive pulmonary disease requiring home oxygen therapy; or reduced functional capacity (<4 METs) or difficult sedation history regardless of modified ASA‐PS class. Functional capacity was estimated clinically based on patient interview and routine physical examination. In such cases, the final determination of the sedation provider was made at the discretion of the individual anesthesiologist based on pre‐procedural clinical history and physical examination, without fixed numerical criteria, within the same institutional risk management committee‐approved framework; additional examinations (e.g., electrocardiography and echocardiography) were performed as clinically indicated.

According to institutional policy, endoscopist‐administered propofol was permitted in patients classified under modified ASA‐PS Class ≤ II. For patients classified under modified ASA‐PS Class III without specific high‐risk features, propofol use by endoscopists was restricted to cases with anesthesiologist approval. Notably, even in patients who did not meet the predefined criteria for high sedation risk, anesthesiologists were consulted at the discretion of the attending endoscopists when there were concerns regarding the safety of endoscopist‐managed sedation, such as in pediatric patients and patients with interstitial pneumonia.

### 2.5 Sedation Management Procedures

For anesthesiologist‐managed sedation, the regimen was determined by anesthesiologists. For endoscopist‐managed sedation, a dedicated endoscopist administered and monitored sedation following the predefined institutional protocol (Figure 1), developed with anesthesiologists and approved by the institutional risk management committee. Sedation was initiated with intravenous pethidine hydrochloride and midazolam, with stepwise reassessment and additional midazolam, as required. Sedation depth was assessed clinically using the Modified Observer's Assessment of Alertness/Sedation (MOAA/S) scale [8], targeting a score of 2–3. Propofol was used only when adequate sedation could not be achieved with midazolam alone and after confirming stable respiratory and hemodynamic status. Endoscopists administering propofol were required to be certified in Immediate Cardiac Life Support, the Japanese Medical Emergency Care Course, or Advanced Cardiovascular Life Support.

**FIGURE 1:**
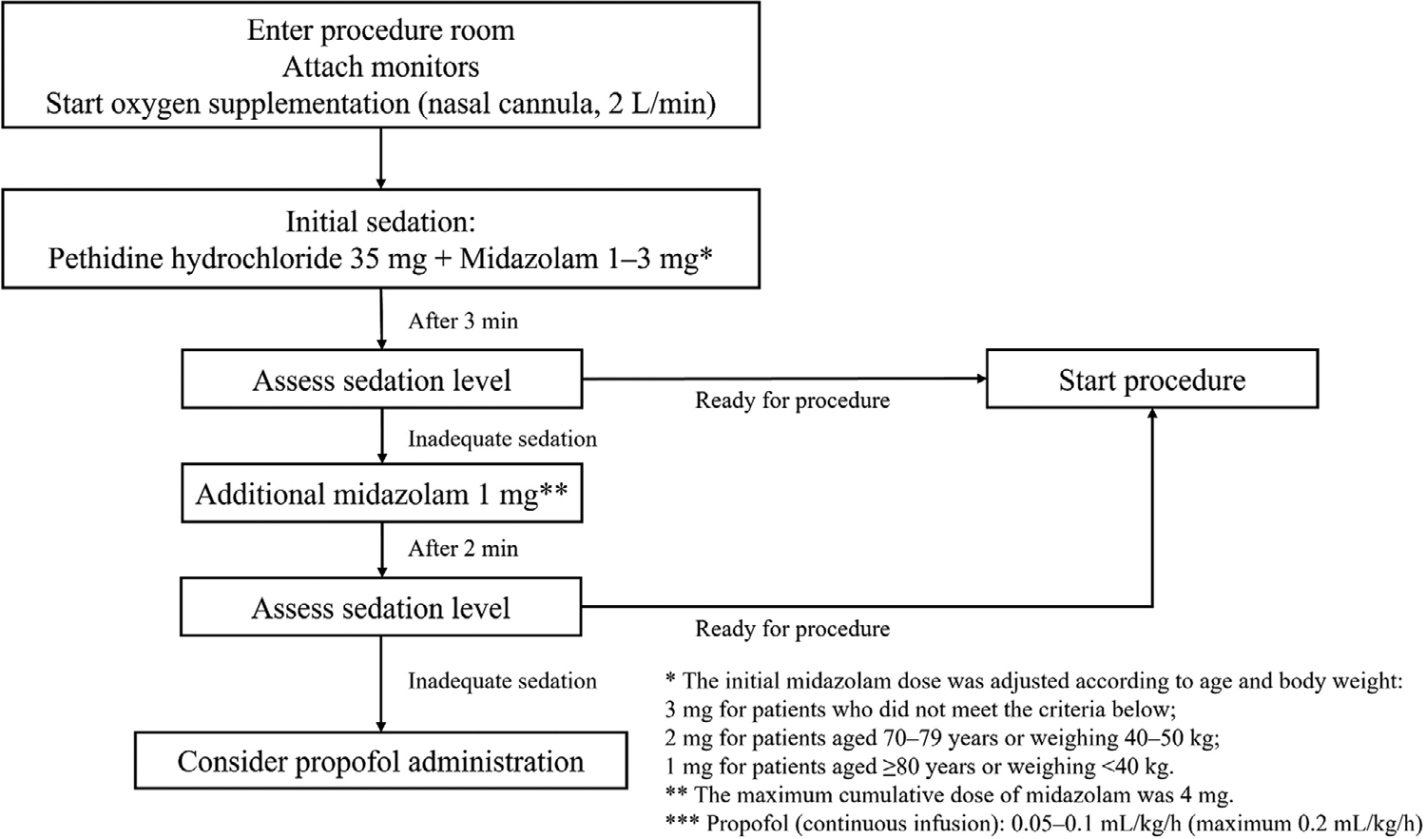
Institutional protocol for endoscopist‐managed sedation. Flowchart of the predefined institutional protocol applied when sedation was induced by endoscopists. Sedation was initiated with intravenous pethidine hydrochloride and midazolam, followed by the stepwise reassessment of sedation depth (MOAA/S score) at predefined intervals. Additional midazolam was administered as needed, and propofol was introduced when adequate sedation could not be achieved with midazolam alone. The protocol was developed in collaboration with anesthesiologists and formally approved by the institutional risk management committee.

### 2.6 Definitions

SRAEs were defined according to a previous report [9] and considered to have occurred when any clinically significant SRAE was observed during the procedure. These events included hypoxemia, the need for airway maneuvers, conversion to general anesthesia, hypotension requiring vasopressor support, cardiac arrhythmias (including tachyarrhythmia and bradyarrhythmia) requiring pharmacological treatment, and interruption or premature termination of sedation. Hypoxemia was defined as SpO_2_ < 90% persisting for >60 s, based on the sentinel adverse event criteria of the World Society of Intravenous Anesthesia [10], to exclude transient desaturation.

Sedation success was defined as completion of the planned endoscopic procedure without SRAEs under endoscopist‐managed sedation; accordingly, the sedation success rate was mathematically equivalent to the complement of SRAE incidence rather than an independent outcome measure. Sedation recovery was evaluated using the modified Aldrete score [11], which was assessed by nursing staff in the dedicated recovery room of the endoscopy center immediately post‐procedure, before the patient was transferred to the ward.

### 2.7 Statistical Analyses

Categorical and continuous variables were compared using Fisher's exact and Mann–Whitney U tests, respectively. To identify SRAE risk factors during endoscopist‐managed sedation, logistic regression analysis was performed. As prespecified in the study protocol, variables that showed statistical significance in univariate analyses were entered into a multivariable logistic regression model using a forward stepwise selection method based on the likelihood ratio. The model was restricted to variables identifiable at the time of pre‐procedural risk assessment, consistent with the study's clinical objective of informing prospective triage decisions. Therefore, intraprocedural variables, including procedure duration, were intentionally excluded, as the analysis was designed to identify pre‐procedural predictors of SRAEs rather than evaluate all potential risk factors comprehensively.

All statistical analyses were performed using BellCurve for Excel (version 4.08; Social Survey Research Information Co., Ltd., Tokyo, Japan) or IBM SPSS Statistics (version 29.0.2.0; IBM Japan, Ltd., Tokyo, Japan). Results with two‐sided *p* < 0.05 were considered significant.

## 3 Results

### 3.1 Patient Characteristics

The patient allocation process based on the modified system is shown in Figure 2. Among the 2000 participants, 231 (11.6%) were classified as having high risk for sedation and 1769 (88.4%) as having non‐high risk. Among the non‐high‐risk patients, 1641 were classified under modified ASA‐PS Class I or II without reduced functional capacity or difficult sedation history, and 128 were classified under Class III without predefined high‐risk features.

**FIGURE 2:**
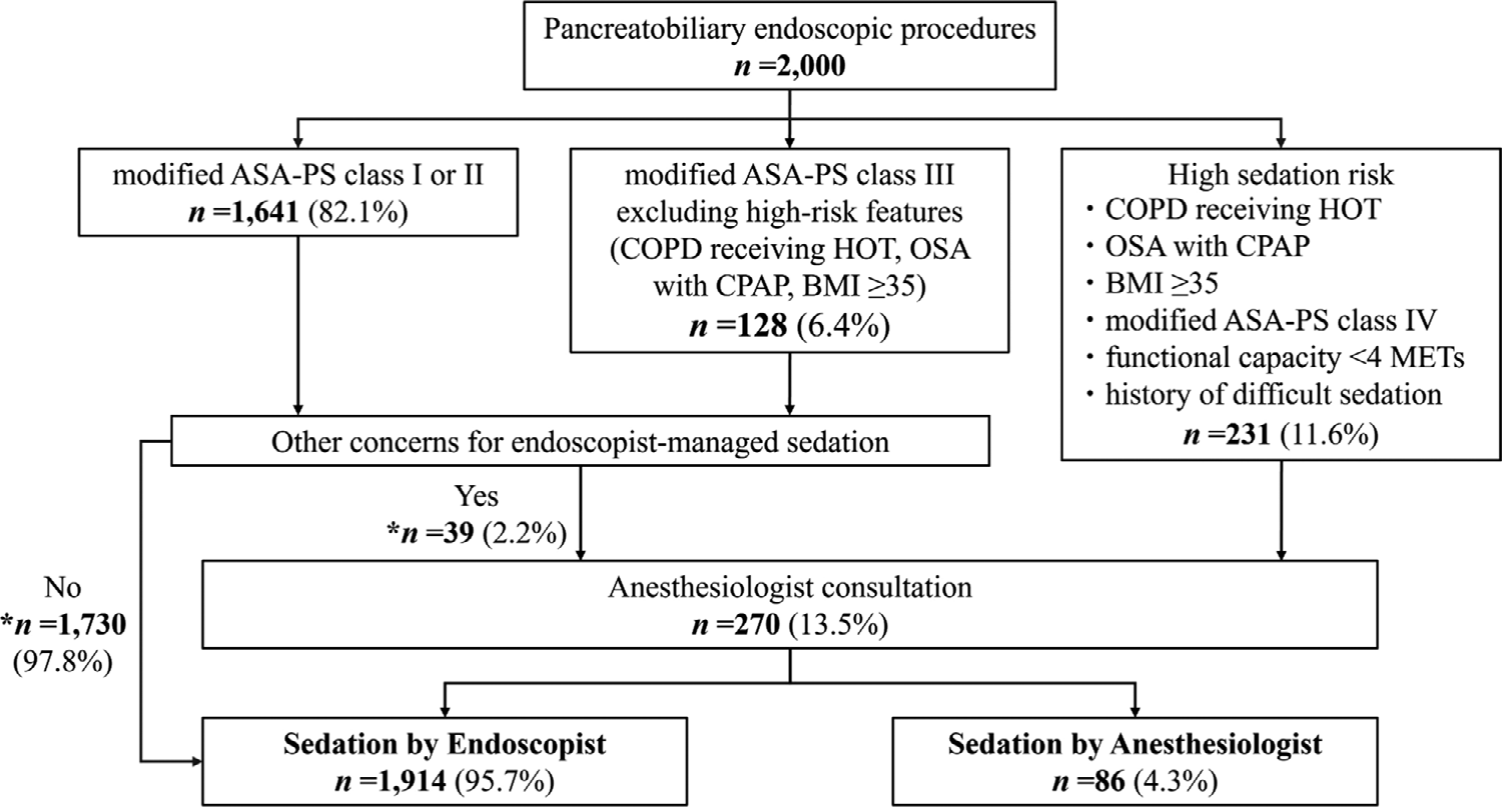
Sedation risk stratification and sedation provider assignment. Flow diagram of sedation risk stratification and sedation provider assignment according to the modified ASA‐PS‐based sedation management system. Patients were classified per the modified ASA‐PS classification combined with predefined sedation‐specific high‐risk clinical factors. Anesthesiologist consultation was performed when indicated, and the final sedation provider was determined following an anesthesiologist assessment. ^*^Percentages are calculated among non‐high‐risk patients (n = 1769); all other percentages in this figure were calculated using the total cohort (n = 2000) as the denominator. Abbreviations: ASA‐PS, American Society of Anesthesiologists Physical Status; BMI, body mass index; COPD, chronic obstructive pulmonary disease; CPAP, continuous positive airway pressure; HOT, home oxygen therapy; METs, metabolic equivalents; OSA, obstructive sleep apnea.

Anesthesiology consultation was performed for 270 patients (231 high‐risk and 39 additional patients referred at endoscopists' discretion, including 10 pediatric patients, all of whom underwent anesthesiologist‐managed sedation; Table S1). Post‐assessment, anesthesiologists and endoscopists managed sedation in 86 (4.3%) and 1,914 (95.7%) patients, respectively. Baseline characteristics are summarized in Table 2. The anesthesiologist‐managed group was younger (63.5 vs. 74 years, *p* < 0.001) and had a higher proportion of benign diseases (76.7% vs. 45.1%, *p* < 0.001) than the endoscopist‐managed group. Proportions of patients classified under modified ASA‐PS Classes III (29.1% vs. 8.1%, *p* < 0.001) and IV (38.4% vs. 2.8%, *p* < 0.001) were higher in the anesthesiologist‐managed group, resulting in a higher proportion of patients classified as having high risk for sedation (72.1% vs. 8.8%, *p* < 0.001). Regarding procedural type, ERCP was more common in the anesthesiologist‐managed group (86.0% vs. 72.5%, *p* = 0.004), whereas EUS‐guided sampling was preferred in the endoscopist‐managed group (23.1% vs. 5.8%, *p* < 0.001).

**TABLE 2:** Patient characteristics.

	All cohort (*n* = 2000)	Sedation by Endoscopist (*n* = 1914)	Sedation by Anesthesiologists (*n* = 86)	*p*‐Value
**Sex, *n* (%)**				0.308
Male	1245 (62.3)	1196 (62.5)	49 (57.0)	
Female	755 (37.7)	718 (37.5)	37 (43.0)	
**Median age, years [IQR]**	74 [63–80]	74 [63–80]	63.5 [41.3–78]	<0.001
**Median BMI, kg/m^2^ [IQR]**	21.7 [19.5–24.1]	21.6 [19.5–24.1]	22.1 [18.7–26.0]	0.455
**Primary disease, *n* (%)**				<0.001
Benign diseases	929 (46.5)	863 (45.1)	66 (76.7)	
Malignant diseases	1071 (53.5)	1051 (54.9)	20 (23.3)	
**Modified ASA‐PS, *n* (%)**				
Class I	734 (36.7)	716 (37.4)	18 (20.9)	0.002
Class II	999 (50.0)	989 (51.7)	10 (11.6)	<0.001
Class III	180 (9.0)	155 (8.1)	25 (29.1)	<0.001
Class IV	87 (4.3)	54 (2.8)	33 (38.4)	<0.001
**High sedation risk, *n* (%)**				<0.001
Yes	231 (11.6)	169 (8.8)	62 (72.1)	
No	1,769 (88.4)	1,745 (91.2)	24 (27.9)	
**High sedation risk** ^a,b^ **(*n* = 231), *n* (%)**				
COPD receiving HOT	13 (5.6)	11 (6.5)	2 (3.2)	0.105
OSA with CPAP	11 (4.8)	11 (6.5)	0	1.000
morbid obesity (BMI ≥35)	20 (8.7)	14 (8.3)	6 (9.7)	<0.001
Modified ASA‐PS class IV	87 (37.7)	54 (32.0)	33 (53.2)	<0.001
Functional Capacity <4 METs	104 (45.0)	84 (49.7)	20 (32.3)	<0.001
History of difficult sedation	31 (13.4)	19 (11.2)	12 (19.4)	<0.001
**Endoscopic procedures, *n* (%)**				
ERCP	1,461 (73.1)	1,387 (72.5)	74 (86.0)	0.004
EUS‐guided sampling	447 (22.3)	442 (23.1)	5 (5.8)	<0.001
EUS‐guided drainage	74 (3.7)	69 (3.6)	5 (5.8)	0.246
Necrosectomy	18 (0.9)	16 (0.8)	2 (2.4)	0.180

^a^ Data are not mutually exclusive; patients may fulfill more than one criterion.

^b^ Percentages are calculated using the corresponding column total as the denominator (all cohort, *n* = 231; endoscopist‐managed group, *n* = 169; anesthesiologist‐managed group, *n* = 62).

Abbreviations: BMI, body mass index; COPD, chronic obstructive pulmonary disease; CPAP, continuous positive airway pressure; ERCP, endoscopic retrograde cholangiopancreatography; EUS, endoscopic ultrasound; HOT, home oxygen therapy; IQR, interquartile range; METs, metabolic equivalents; OSA, obstructive sleep apnea.

### 3.2 Sedation Outcomes in the Endoscopist‐managed Group

The sedation outcomes in the 1,914 patients managed by endoscopists are presented in Table 3. SRAEs occurred in 63 patients (3.3%; 95% confidence interval [CI], 2.6%–4.2%), corresponding to a sedation success rate of 96.7%. Propofol was administered in 914 cases (47.8%). The median total sedation time was 34 (interquartile range [IQR], 25–47), and the median modified Aldrete score after the procedure was 9 (IQR, 9–10). Rapid Response Team activation was required in only two cases (0.1%), both owing to persistent hypoxemia. These events were managed with airway support and supplemental oxygen, and neither case required tracheal intubation or transfer to the intensive care unit.

**TABLE 3:** Sedation outcomes by endoscopists.

*n* = 1914	
**Median sedation time, min [IQR]**	
ERCP	36 [26–50]
EUS‐guided sampling	27 [21–35]
EUS‐guided drainage	43 [33–57]
Necrosectomy	64 [53.4–68.5]
All	34 [25–47]
**Use of propofol, *n* (%)**	
Yes	914 (47.8)
No	1000 (52.2)
**SRAEs, *n* (%)**	
Yes	63 (3.3)
No	1851 (96.7)
**Requirement for Rapid Response Team, *n* (%)**	
Yes	2 (0.1)
No	1912 (99.9)
**Sedation success rate**	96.7%
**Median modified Aldrete score, [IQR]**	9 [9–10]

Abbreviations: ERCP, endoscopic retrograde cholangiopancreatography; EUS, endoscopic ultrasound; IQR, interquartile range; SRAEs, sedation‐related adverse events.

### 3.3 SRAEs Details

SRAE distribution among 63 affected patients is presented in Table 4. The most frequent event was hypotension requiring vasopressor administration (2.1%), followed by cardiac arrhythmia necessitating pharmacologic treatment (0.6%) and hypoxemia (0.5%). Airway support was needed in 0.1% of cases. No patient required conversion to general anesthesia. Sedation was interrupted or prematurely terminated in four patients (0.2%; Table 4), corresponding to a procedure completion rate of 99.8%.

**TABLE 4:** Details of sedation‐related adverse events.

*n* = 63^a^	No. of patients (%)
Hypoxemia (SpO_2_ < 90%)	9 (0.5)
Requirement of airway maneuvers	2 (0.1)
Conversion to general anesthesia	0
Hypotension requiring vasopressors	41 (2.1)
Cardiac arrhythmia requiring medication	11 (0.6)
Interruption or termination of sedation	4 (0.2)

^a^ Data are not mutually exclusive; patients may fulfill more than one criterion.

Abbreviation: SpO_2_, peripheral oxygen saturation.

### 3.4 SRAE Risk Factors

SRAE risk factors were analyzed in the 1914 patients managed by endoscopists. In the univariate analysis, ERCP was significantly associated with an increased SRAE risk (odds ratio [OR] = 2.054, 95% CI: 1.037–4.068; *p* = 0.039), whereas EUS‐guided sampling was associated with a reduced risk (OR = 0.407, 95% CI: 0.184–0.899; *p* = 0.026). No other patient‐ or procedure‐related variables were significantly associated with SRAEs (Table 5). In the multivariable logistic regression analysis, only EUS‐guided sampling remained independently associated with a reduced SRAE risk (OR = 0.407, 95% CI: 0.184–0.899, *p* = 0.026). No independent risk factor was associated with increased SRAE incidence (Table 5).

**TABLE 5:** Pre‐procedural risk factors for sedation‐related adverse events.

	SRAEs (+) (*n* = 63)	SRAEs (‐) (*n* = 1851)	Univariate analysis		Multivariate analysis	
			OR [95% CI]	*p*‐value	OR [95% CI]	*p*‐value
Age			1.021 [0.998–1.044]	0.072		
Men	40	1156	1.046 [0.621–1.761]	0.867		
Malignant diseases	35	1016	1.027 [0.620–1.703]	0.917		
BMI			1.008 [0.941–1.079]	0.821		
Modified ASA‐PS Class III	1	154	0.178 [0.024–1.291]	0.088		
Modified ASA‐PS Class IV	3	51	1.765 [0.536–5.815]	0.351		
COPD receiving HOT	0	11	n.c.			
OSA with CPAP	0	11	n.c.			
morbid obesity (BMI ≥35)	0	14	n.c.			
Functional capacity <4 METs	5	79	1.934 [0.755–4.955]	0.170		
History of difficult sedation	1	18	1.642 [0.216–12.500]	0.632		
High sedation risk	8	161	1.527 [0.715–3.262]	0.275		
ERCP	53	1334	2.054 [1.037–4.068]	0.039	n.e.	
EUS‐guided sampling	7	435	0.407 [0.184–0.899]	0.026	0.407 [0.184–0.899]	0.026
EUS‐guided drainage	1	68	0.423 [0.058–3.095]	0.397		
Necrosectomy	2	14	4.302 [0.957–19.346]	0.057		

BMI, body mass index; CI, confidence interval; COPD, chronic obstructive pulmonary disease; CPAP, continuous positive airway pressure; ERCP, endoscopic retrograde cholangiopancreatography; EUS, endoscopic ultrasound; HOT, home oxygen therapy; METs, metabolic equivalents; OR, odds ratio; OSA, obstructive sleep apnea; n.c., not calculable; n.e., not evaluable.

## 4 Discussion

Here, SRAEs occurred in only 3.3% of patients managed by endoscopists. Akhter et al. [12] reported comparable safety between moderate sedation administered without dedicated anesthesia personnel and anesthesiologist‐assisted sedation during ERCP. However, their sedation strategy was determined by an endoscopist, and no predefined multifactorial system incorporating structured risk stratification and formal anesthesiologist involvement was prospectively implemented. The SRAE incidence in our study was within the range of component event rates (hypoxemia 6.0% and hypotension 1.8%) reported for propofol‐based biliopancreatic endoscopy in Japan [13]. However, direct comparisons are limited by differences in outcome definitions and study design. These findings suggest that the two‐step process—risk stratification based on the modified ASA‐PS classification followed by anesthesiologist assessment—contributes to a low SRAE incidence during endoscopist‐managed sedation in routine practice. However, the relative contribution of each component cannot be determined from our single‐arm design.

Japan's limited anesthesiology resources preclude uniform implementation of advanced ASA‐PS classifications; the present system addresses this by prospectively identifying patients who require anesthesiologists, representing a pragmatic model for Japanese practice.

ASA‐PS classification was originally designed for epidemiologic reporting rather than precise prediction of individual perioperative risk. Horvath et al. [14] highlighted limitations including interobserver variability and insufficient consideration of factors such as frailty and sedation tolerance. The ASGE guidelines similarly recommend incorporating airway assessment and sedation history alongside ASA‐PS documentation [5]. The modified ASA‐PS system in the present study was developed with anesthesiologists to better reflect the clinical context of endoscopic sedation in Japanese patients, simplifying certain descriptive elements while emphasizing factors more directly relevant to sedation risk.

Our two‐tiered structure—combining modified ASA‐PS classification with explicitly defined high‐risk criteria and direct pre‐anesthetic evaluation, including airway assessment—possibly contributed to the favorable safety outcomes observed.

In the multivariable analysis, only EUS‐guided sampling was independently associated with reduced SRAE incidence. To support the robustness of this model, we performed a supplementary sensitivity analysis comparing alternative variable‐selection strategies (Table S2). ERCP, which was significant in the univariate analysis, could not be evaluated in the multivariable model. This was attributable to quasi‐complete separation arising from its near‐complementary relationship with EUS‐guided sampling (patients undergoing ERCP rarely underwent EUS‐guided sampling, and vice versa), such that only EUS‐guided sampling was retained by the stepwise selection procedure, and its multivariable estimate coincided with the univariate estimate (Table S2). As a sensitivity analysis, we additionally included all five variables with univariate P<0.1 (including age) into a separate model regardless of statistical significance; none reached significance, supporting the absence of an independent predictor of SRAEs (Table S2). Because procedure duration was not included as a covariate in the multivariable model, this explanation cannot be statistically substantiated by the present data and should be regarded as hypothesis‐generating. We note descriptively that EUS‐guided sampling had a shorter median sedation time than other procedures (27 vs. 34 min overall) and that prolonged procedure time has been reported as a risk factor for SRAEs [15, 16]. Additionally, EUS‐guided sampling was the only procedure performed in the left lateral position, whereas all other procedures were performed in the prone position, and this difference in position cannot be distinguished from procedure type itself as a potential explanation for the lower SRAE risk observed with EUS‐guided sampling. Because sedation duration is an intraprocedural outcome rather than a pre‐procedural variable, it was not entered into the multivariable model, which was restricted to factors identifiable before the procedure began. Consequently, this association should not be interpreted as evidence that EUS‐guided sampling itself confers protection against SRAEs but rather as a reflection of its typically shorter and more predictable procedural course. Notably, previously reported risk factors, including advanced age, high body mass index, obstructive sleep apnea, and high ASA‐PS class [5, 17, 18, 19, 20], were not independent predictors, possibly reflecting selective allocation of high‐risk patients to anesthesiologist‐managed sedation.

This study has some limitations. First, it was conducted at a single center, and the institutional policy restricting endoscopist‐administered propofol in modified ASA‐PS Class III patients to cases approved by anesthesiologists may not be applicable elsewhere. Functional capacity was also assessed clinically rather than by formal exercise testing, without evaluation of inter‐rater reliability. Second, hypoxemia was defined as SpO_2_ < 90% persisting for ≥60 s. Although this definition was intended to capture clinically meaningful events, transient and mild desaturation episodes may have been excluded, potentially leading to the underestimation of the overall SRAE incidence. Third, this was a single‐arm study without a concurrent comparison group. Consequently, the favorable safety outcomes observed cannot be directly attributed to the modified ASA‐PS‐based sedation management system itself; they may instead reflect other factors, including institutional experience, structured sedation protocol, and exclusion of the highest‐risk patients through anesthesiologist triage. Therefore, this study should be regarded as a single‐center study demonstrating the feasibility of implementing an institutional sedation triage system, rather than as a confirmatory evaluation of its clinical effectiveness. Fourth, the inter‐rater reliability of the modified ASA‐PS classification system and its concordance with the standard ASA‐PS classification system were not evaluated, because this was not a prespecified study objective. Multicenter studies assessing inter‐rater reliability and external validation are warranted to establish generalizability beyond our institution. Finally, although sedation depth was targeted to an MOAA/S score of 2–3, the actual intraprocedural MOAA/S scores achieved were not systematically recorded, and only post‐procedural modified Aldrete scores were available. The absence of intraprocedural sedation‐depth data limits our ability to directly relate the sedation depth achieved to SRAE occurrence.

## 5 Conclusions

This prospective feasibility study demonstrated that a two‐step triage system, combining modified ASA‐PS‐based classification with anesthesiologist assessment, could be implemented in routine pancreatobiliary endoscopic practice: 95.7% of patients were managed by endoscopists, with an SRAE incidence of 3.3% and a procedure completion rate of 96.7%. Because the final sedation‐provider decision relied on anesthesiologist discretion rather than fixed criteria, and because no comparison group was included, these findings do not demonstrate that the system improves sedation safety. Multicenter or comparative validation is warranted before wider adoption of the system.

## Author Contributions

**Taro Hanaoka**: Investigation (lead); Formal analysis (lead); Writing – original draft preparation (lead); Writing – review & editing (equal); Visualization (lead). **Kosuke Okuwaki**: Conceptualization (lead); Methodology (lead); Investigation (supporting); Supervision (lead); Project administration (lead); Writing – review & editing (equal). **Masafumi Watanabe**: Methodology (supporting); Investigation (supporting); Formal analysis (supporting); Writing – review & editing (equal). **Hiromi Matsuda**: Methodology (supporting); Writing – review & editing (equal). **Kai Adachi**: Investigation (supporting); Writing – review & editing (equal). **Akihiro Tamaki**: Investigation (supporting); Writing – review & editing (equal). **Daisuke Ogawa**: Investigation (supporting); Writing – review & editing (equal). **Mayuko Sakai**: Methodology (supporting); Writing – review & editing (equal). **Chika Kusano**: Methodology (supporting); Supervision (supporting); Writing – review & editing (equal). All authors approved the final version of the manuscript and agree to be accountable for all aspects of the work.

## Funding

The authors have nothing to report.

## Ethics Statement

This study was conducted per the tenets of the Declaration of Helsinki. The study was approved by the Institutional Review Board for Clinical Research and Treatment of Kitasato University Hospital (approval number: B23‐122).

## Consent

The requirement for written informed consent was waived by the Institutional Review Board. However, verbal consent was obtained from the patients and documented. Study information was also disclosed via an opt‐out approach on the hospital website, and participants could withdraw at any time without affecting their clinical care.

## Conflicts of Interest

The authors declare no conflicts of interest.

## Supporting information




**TABLE S1** Reasons for anesthesiologist consultation among the 39 patients referred at endoscopists' discretion despite not meeting the predefined high‐risk criteria. Reasons for anesthesiologist consultation among the 39 patients referred at endoscopists' discretion despite not meeting the predefined high‐risk criteria, grouped by clinical category.

**TABLE S2** Sensitivity analyses of the multivariable logistic regression model for sedation‐related adverse events. Sensitivity analyses of the multivariable logistic regression model for sedation‐related adverse events, comparing forward stepwise selection (the model reported in the main text) with forced‐entry models, to assess the robustness of the association between EUS‐guided sampling and reduced SRAE risk.

## Data Availability

The data that support the findings of this study are available from the corresponding author on reasonable request.
