# Supplementary material for: Evaluating a Modified American Society of Anesthesiologists Physical Status‐Based Sedation Management System for Pancreatobiliary Endoscopy: A Prospective Feasibility Study
Source: DEN Open. 2026 Oct 12;7(1):e70445. doi: 10.1002/deo2.70445 (PMC13658845; doi:10.1002/deo2.70445)
Supplement: Supplementary file 1 [file DEO2-7-e70445-s001.docx]

**Table S2. Sensitivity analyses of the multivariable logistic regression model for sedation-related adverse events**

|  | **Model A (reported in manuscript; forward stepwise selection)** | | | **Model B (forced entry, for comparison)** | | | **Model C (forced entry of all variables with univariate P<0.1)** | | |
| --- | --- | --- | --- | --- | --- | --- | --- | --- | --- |
| **Variable** | **OR** | **95% CI** | **P-value** | **OR** | **95% CI** | **P-value** | **OR** | **95% CI** | **P-value** |
| Age (per 1-year increase) | – | – | – | – | – | – | 1.019 | 0.997–1.043 | 0.096 |
| Male sex | – | – | – | – | – | – | – | – | – |
| Malignant disease | – | – | – | – | – | – | – | – | – |
| BMI (per 1 kg/m²) | – | – | – | – | – | – | – | – | – |
| modified ASA-PS Class III | – | – | – | – | – | – | 0.172 | 0.024–1.251 | 0.082 |
| modified ASA-PS Class IV | – | – | – | – | – | – | – | – | – |
| COPD receiving HOT | – | – | – | – | – | – | – | – | – |
| OSA with CPAP | – | – | – | – | – | – | – | – | – |
| Morbid obesity (BMI ≥35) | – | – | – | – | – | – | – | – | – |
| Functional capacity <4 METs | – | – | – | – | – | – | – | – | – |
| History of difficult sedation | – | – | – | – | – | – | – | – | – |
| High sedation risk | – | – | – | – | – | – | – | – | – |
| ERCP | n.e. |  |  | 1.086 | 0.332–3.550 | 0.891 | 2.563 | 0.348–18.856 | 0.355 |
| EUS-guided sampling | 0.407 | 0.184–0.899 | 0.026 | 0.440 | 0.111–1.736 | 0.241 | 1.082 | 0.131–8.950 | 0.942 |
| EUS-guided drainage | – | – | – | – | – | – | – | – | – |
| Necrosectomy | – | – | – | – | – | – | 11.355 | 0.945–136.391 | 0.055 |

Model A: Forward stepwise selection (likelihood ratio) among the two variables that were significant in univariate analysis (ERCP, EUS-guided sampling), as prespecified in the study protocol; this is the model reported in the main manuscript (Table 5). Model B: Forced entry of the same two variables, shown to illustrate their collinearity. Model C: Forced entry of all five variables with univariate P<0.1 (age, modified ASA-PS Class III, ERCP, EUS-guided sampling, necrosectomy), performed as a sensitivity analysis to confirm that clinically important variables (e.g., age) did not become significant independent predictors when entered regardless of univariate significance.

In Model B, neither ERCP nor EUS-guided sampling reached significance when forced into the model together, indicating collinearity between the two variables (patients undergoing ERCP rarely underwent EUS-guided sampling, and vice versa). In the stepwise selection procedure used for Model A, only EUS-guided sampling was retained, and ERCP could not be evaluated (n.e.); the retained variable's estimate coincided with its univariate estimate because it was the sole predictor in the final model. In Model C, none of the five variables, including age, reached statistical significance, supporting the conclusion of the primary analysis (Model A) that no independent predictor of SRAEs was identified.

OR, odds ratio; CI, confidence interval; BMI, body mass index; ASA-PS, American Society of Anesthesiologists Physical Status; COPD, chronic obstructive pulmonary disease; HOT, home oxygen therapy; OSA, obstructive sleep apnea; CPAP, continuous positive airway pressure; METs, metabolic equivalents; ERCP, endoscopic retrograde cholangiopancreatography; EUS, endoscopic ultrasound; n.e., not evaluable (excluded by the stepwise selection procedure).
