# Supplementary material for: Evaluating a Modified American Society of Anesthesiologists Physical Status‐Based Sedation Management System for Pancreatobiliary Endoscopy: A Prospective Feasibility Study
Source: DEN Open. 2026 Oct 12;7(1):e70445. doi: 10.1002/deo2.70445 (PMC13658845; doi:10.1002/deo2.70445)
Supplement: Supplementary file 2 [file DEO2-7-e70445-s002.docx]

**Table S1. Reasons for anesthesiologist consultation among the 39 patients referred at endoscopists' discretion despite not meeting the predefined high-risk criteria**

| **Category** | **n** |
| --- | --- |
| Pediatric | 10 |
| Respiratory disease | 11 |
| Cardiovascular disease | 13 |
| Hepatic disease | 2 |
| Other | 3 |
| **Total** | **39** |

Categories were defined as follows: Pediatric, patients under 18 years of age; respiratory disease, cardiovascular disease, and hepatic disease, based on the endoscopist's clinical judgment of disease severity; other, reasons not classifiable into the above categories. All 10 pediatric patients underwent anesthesiologist-managed sedation.
